# Oral potentially malignant disorders: clinical-pathological study of 684 cases diagnosed in a Brazilian population

**DOI:** 10.4317/medoral.23197

**Published:** 2019-12-24

**Authors:** Fábio Ramôa Pires, Maria Eduarda Zeraik Barreto, Jéssica Gomes Rodrigues Nunes, Natália Santos Carneiro, Alexandro Barbosa de Azevedo, Teresa Cristina Ribeiro Bartholomeu dos Santos

**Affiliations:** 1 PhD, Oral Pathology, School of Dentistry, State University of Rio de Janeiro, Rio de Janeiro, RJ, Brazil; 2 DDS, School of Dentistry, State University of Rio de Janeiro, Rio de Janeiro, RJ, Brazil; 3 DDS, Oral Pathology, Piracicaba Dental School, University of Campinas, Piracicaba, SP, Brazil; 4 MSc, Oral Pathology, School of Dentistry, State University of Rio de Janeiro, Rio de Janeiro, RJ, Brazil.

## Abstract

**Background:**

The frequency and distribution of oral potentially malignant disorders (OPMD) may vary among different populations. The aim of the present study was to evaluate the clinical-pathological characteristics of OPMD diagnosed in a Brazilian oral pathology laboratory over a period of 11 years.

**Material and Methods:**

All cases diagnosed as leukoplakia, speckled leukoplakia, erythroplakia, and actinic cheilitis from 2005 to 2015 were analyzed. Clinical information was obtained from laboratory forms and histological information was obtained from histological slides stained with hematoxylin and eosin.

**Results:**

the final sample was comprised of 684 cases, of which 292 were males and 392 were females. The mean age was 58 years. The anatomical site most often involved was the lateral border of the tongue (23%), followed by the lower lip (20%), and by the buccal mucosa/vestibule (18%). Leukoplakia accounted for 82% of the sample (564 cases). The mean size of the leukoplakia and speckled leukoplakia lesions was 13 mm (ranging from 1 to 100 mm) and 15 mm (ranging from 5 to 30 mm), respectively (*p*=0.460). Males reported smoking and drinking alcohol more frequently than females (*p*=0.001 and *p* <0.0001, respectively). In half of the cases, dysplasia was not histologically detected, while slight dysplasia was detected in 28% of the cases. The lesions from patients aged from 41 to 80 years presented moderate and severe dysplasia more often than lesions from patients in other age groups.

**Conclusions:**

OPMD were more common in females in their sixties. Females were more frequently affected in all anatomical sites, except for the lips. Leukoplakia lesions were the most common OPMD, followed by actinic cheilitis. The lateral border of the tongue was the most affected anatomical site. OPMD located in the floor of the mouth/sublingual mucosa and lesions from older patients presented severe epithelial dysplasia with greater frequency.

** Key words:**Potentially malignant disorders, oral, leukoplakia, speckled leukoplakia, actinic cheilitis, squamous cell carcinoma.

## Introduction

Oral potentially malignant disorders (OPMD) are tissue changes that may precede squamous cell carcinoma (SCC) (1-3), a malignant neoplasm that accounts for 80-90% of all cancers in the oral cavity. The most common disorders in this group are leukoplakia, speckled leukoplakia, erythroplakia, and actinic cheilitis (3-7). Considering the various characteristics of OPMD in different populations, knowledge of their clinical-pathological profile is an important diagnostic tool and, therefore, may play a role in preventing malignant transformation of these lesions (1,2,8-10). Given the low number of epidemiological studies about this topic in the Brazilian population, this study aimed to analyze the clinical-pathological features of OPMD diagnosed in a Brazilian oral pathology center over a period of 11 years.

## Material and Methods

Files describing all cases classified as OPMD (leukoplakia, speckled leukoplakia, erythroplakia, and actinic cheilitis) from the University of Rio de Janeiro's Oral Pathology Laboratory from 2005 to 2015 (an 11-year period) were reviewed for the purposes of this study. Laboratory records lacking clinical information, with poorly representative specimens, or in which the OPMD diagnosis could not be confirmed (for example, specimens presenting acanthosis and hyperkeratosis, where the possibility of frictional keratosis could not be dismissed) were excluded from the analysis.

The clinical information obtained from the laboratory files included: age and sex of the patient, anatomical location, size and clinical aspect of the lesion (leukoplakia, erythroplakia, speckled leukoplakia, ulceration, and others), and whether the patient reported smoking or drinking. Histological slides stained with hematoxylin and eosin were reviewed and the degree of dysplasia was evaluated according to previously described criteria (3,11,12). Lesions were classified as showing no dysplasia, mild dysplasia, moderate dysplasia, or severe dysplasia.

Data were tabulated and analyzed descriptively and comparatively using chi-square and T student tests through SPSS (Statistical Program for Social Sciences version 2.0), with a significance level of 5% (*p*<0.05). The study was approved by the Ethics in Research Committee, State University of Rio de Janeiro, under the protocol number 04187318.7.0000.5259.

## Results

The final sample was comprised of 684 OPMD diagnosed in 392 females (57%) and 292 males (43%). The mean age of the patients was 58 years (ranging from 7 to 100 years), with statistically significant difference between the mean age of male patients (55 years, ranging from 7 to 89 years) and the mean age of female patients (60 years, ranging from 8 to 100 years) (*p*<0.001). Eighty-two percent of the patients were between 41 and 80 years old.

The lateral border of the tongue was the most frequently affected anatomical site (23%), followed by the lower lip (20%), buccal mucosa/vestibule (19%), mandibular alveolar mucosa (11%), and maxillary alveolar mucosa (10%). Leukoplakia represented 82% of the sample (564 cases), followed by actinic cheilitis (78 cases, 12%), and speckled leukoplakia (42 cases, 6%). Pure erythroplakias were not found in the sample. Ulceration was described in 66 cases (10%). The mean size of the leukoplakias was 13 mm (ranging from 1 to 100 mm) and the mean size of speckled leukoplakias was 15 mm (ranging from 5 to 30 mm) (*p*=0.460). With regard to smoking and drinking, 53% of the patients were smokers/ex-smokers and 30% were drinkers. Approximately 63% of the males and 46% of the females reported smoking (*p*=0.001), and approximately 48% of the males and 15% of the females reported drinking (*p*<0.0001). Histological analysis showed that 337 cases (49%) showed no dysplasia, and 188 cases (28%), 83 cases (12%), and 76 cases (11%) showed mild, moderate, and severe epithelial dysplasia, respectively.

The anatomical distribution of lesions showed statistically significant differences according to the sex of the patient (*p*<0.0001). The number of females affected was greater in all anatomical sites, except for the lips (Table 1).

**Table 1:**
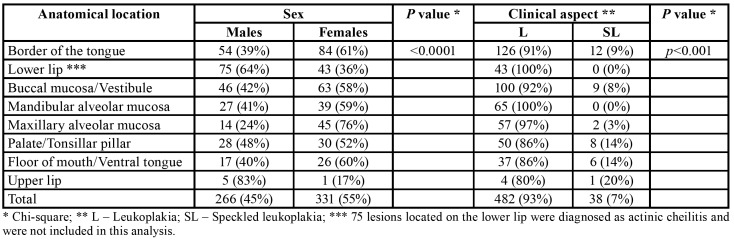
Anatomical distribution of the lesions according with the sex of the patients and clinical aspect of the lesions.

The mean age of the patients did not show statistically significant difference with regard to the anatomical location of the lesions (*p*=0.207). The anatomical distribution of the lesions according to the final diagnosis showed statistically significant difference between leukoplakia and speckled leukoplakia (*p*<0.001) (Table 1).

Of the 292 OPMD diagnosed in male patients, 220 (75%) were leukoplakias, 19 (7%) were speckled leukoplakias, and 53 (18%) were actinic cheilitis. In female patients, 344 cases (88%) were diagnosed as leukoplakia, 23 (6%) as speckled leukoplakia, and 25 (6%) as actinic cheilitis, with statistically significant difference between female and male patients (*p*<0.001). Leukoplakia, speckled leukoplakia, and actinic cheilitis presented ulceration in 9%, 10%, and 18% of the cases, respectively (*p*=0.03).

The distribution of the different degrees of dysplasia showed statistically significant difference between the various anatomical sites were the lesions were found (*p*=0.002). The floor of the mouth and ventral tongue were the anatomical sites with the greatest percentage of cases with moderate and severe dysplasia (Table 2). Leukoplakias and speckled leukoplakias presented moderate or severe dysplasia in 15% and 41% of the cases, respectively (*p*<0.0001). The distribution of the different degrees of dysplasia among the various age groups showed that patients aged between 41-60 years, 61-80 years, and older than 80 years presented moderate and severe dysplasia in 24%, 27%, and 30% of the lesions, respectively, contrasting with the patients younger than 40 years old (*p*<0.001) (Table 3).

**Table 2:**
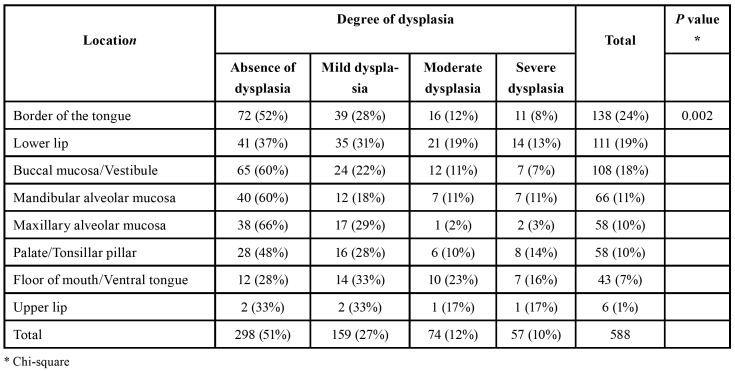
Anatomical distribution of the lesions according to their degree of dysplasia.

**Table 3:**
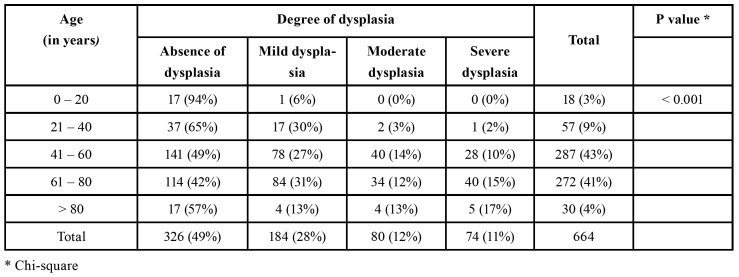
Distribution of the different degrees of dysplasia among the various patient age groups.

## Discussion

OPMD are relatively common, with a worldwide prevalence of 4.4%, while leukoplakia alone has a prevalence of 4.1% (13). Numerous studies on OPMD have been conducted worldwide in the past few years; some of these studies focused on Brazilian populations (10). Knowledge of the social-demographic profile of patients with OPMD in a given population is important for understanding the most prevalent risk factors and for outlining prevention and early diagnosis strategies. In the present study, female patients made up 57% of the sample. The predominance of females has also been observed in previous studies on Brazilian populations (14,15) and in other worldwide studies (9,16); however, this predominance was not observed in several studies involving different populations (1,2,7,8,13).

One possible explanation for the higher frequency of females in our sample may be the growing number of women who smoke and drink alcohol. Consumption of tobacco and alcohol is widespread, and the synergistic effect of these two behaviors appears to be the greatest risk factor for development of OPMD and oral cancers worldwide (2,15,17). Still, in the present sample, the percentage of female smokers and drinkers was lower than the percentage of male smokers and drinkers. Another potential explanation for the greatest frequency of OPMD diagnosed in females may result from the fact that women tend to seek oral medicine services more often than men, increasing the odds that lesions in women would be diagnosed and treated.

Over 80% of the patients were aged between 41 and 80 years, which highlights the connection between age and increased risk for developing OPMD, as previously reported in the scientific literature (1,2,9,13-16,18). It is important to note that the age distribution observed in the present study was similar to a descriptive study that evaluated 346 cases of SCC diagnosed by the same laboratory between 2005 and 2012 (19). The mean age of male patients diagnosed with OPMD in our study was lower than the age of their female counterparts, which is in agreement with the findings from the SCC study described above (19). In our study, the age of the patients did not show statistically significant difference with regard to the anatomical site where the lesions were found.

Leukoplakias are the most common oral OPMD, with a worldwide incidence between 2 and 4% (2,13). In the present study, 82% of the patients had a diagnosis of leukoplakia, similarly to a previous study on a Brazilian population (18). None of the cases in our sample were diagnosed as pure erythroplakia, possibly due to the lack of precise clinical correlation required for a lesion to be classified as pure erythroplakia. Cases of oral lichen planus were excluded from our sample, due to the difficulties in distinguishing its clinical features from other OPMD. Some earlier studies have included cases diagnosed as dysplastic oral lichen planus, but lacked detailed individual histological information to document the malignant transformation process in those lesions (16).

In the present sample, leukoplakia and speckled leukoplakia were more frequent in females in all anatomical sites, except for the lips. As expected, actinic cheilitis was more common in male patients (68%), due to the higher frequency of occupational exposure to ultraviolet radiation and to the lower likelihood of sunscreen use among men (6,20). Actinic cheilitis, if undiagnosed and untreated, may evolve into an ulcerated lesion; the results from the present study confirm this fact, since 18% of the cases presented ulceration (almost twice the number of ulcerated leukoplakia and speckled leukoplakia lesions). Furthermore, the frequency of moderate/severe dysplasia in lesions of the lower lip was greater than the frequency of moderate/severe dysplasia in most of the other anatomical sites, which corroborates the findings from previous studies (6).

Comparison between the mean size of leukoplakias and of speckled leukoplakias did not show statistically significant difference. This finding suggests that the presence of erythroplakia does not appear to be part of the natural evolution of OPMD. Therefore, the size of the lesion alone is not an indicator of its potential for malignant transformation. Nonetheless, Speight *et al*. (21) listed lesion size greater than 200 mm2 as a clinical parameter associated with increased risk of malignant transformation in OPMD.

The tongue – especially the lateral border – was the most frequently affected anatomical site, a finding that is in agreement with other studies (16). This site was also most frequently affected by SCC, according to an earlier study carried out with the same population (19). Other studies, however, reported greater frequencies of buccal mucosa (9) or alveolar mucosa involvement (14,18). This may be due to population variations, difficulty in distinguishing leukoplakia from other lesions (for example, reactional hyperkeratosis) clinically or pathologically, or even inclusion of oral lichen planus lesions in the sample.

The degree of dysplasia is based on structural and cytological characteristics of the epithelium and is one of the findings commonly used to evaluate the risk of malignant transformation in OPMD (4,5,11,12,14,16,21). Half the lesions included in this study showed no epithelial dysplasia on histological analysis but were still considered OPMD as the clinical aspect was compatible with leukoplakias or leukoerythroplakias and they could not be diagnosed as any other oral lesions. In the present study, comparison of the anatomical location and degree of dysplasia showed that most of the lesions found on the lateral border of the tongue either did not present dysplasia or only presented mild dysplasia (52% and 28% of the cases, respectively.) The floor of the mouth and ventral tongue were the anatomical sites with the greatest percentage of moderate (23%) and severe dysplasia (16%). These results are in agreement with previous studies on a Brazilian population (14) and reinforce the notion that OPMD located on the tongue and on the floor of the mouth have higher risk of malignant transformation (21). Accordingly, these anatomical sites deserve special attention because they are the most commonly affected by squamous cell carcinoma (SCC) in this population (19). The frequency of severe epithelial dysplasia increased as patient age increased; no correlation with age was observed for lesions with mild or moderate dysplasia. This finding supports the theory that the malignant transformation process is slow and gradual over the years, and that older patients are at greater risk for malignant transformation than their younger counterparts (1,17,21). In addition, analysis of the degree of dysplasia based on the clinical aspect of the lesions revealed that leukoplakias and speckled leukoplakias presented moderate or severe dysplasia in 15% and 41% of the cases, respectively (*p*<0.0001), similarly to the findings from previous studies (13,14,18).

In conclusion, in the Brazilian population sample from the present study, most OPMD were diagnosed in female patients in their 60s. Females were more frequently affected by lesions found in all anatomical sites, except for the lips. Leukoplakias were the most commonly observed OPMD, followed by actinic cheilitis. The lateral border of the tongue, the lower lip, and the buccal mucosa/vestibule were the most frequently affected anatomical sites. Lesions found on the floor of the mouth/ventral tongue presented the highest frequency of severe epithelial dysplasia, and the highest frequency of severe epithelial dysplasia was observed in older patients.
